# Clinical trial capacity in African primary care: Advancing contextualised implementation

**DOI:** 10.4102/phcfm.v17i1.5158

**Published:** 2025-11-06

**Authors:** Schawanya K. Rattanapitoon, Nav La, Jun Norkaew, Nathkapach K. Rattanapitoon

**Affiliations:** 1 Department of Family Medicine and Occupational Medicine, FMC Medical Center of Thailand, Nakhon Ratchasima, Thailand; 2 Faculty of Medicine, International University, Phnom Penh, Cambodia; 3 Faculty of Public Health, Vongchalitkul University, Nakhon Ratchasima, Thailand

## Responding to Mash et al.: Urgency, equity, and pragmatism in African primary care trials

We commend Mash and colleagues^1^ for their timely and thorough exposition of clinical trial methodology, tailored to the realities of family medicine and primary care. Their article not only highlights the critical underutilisation of randomised controlled trials in low- and middle-income countries – particularly in African primary care but also provides an incisive roadmap for methodological advancement. We write to reinforce and expand their recommendations, arguing that contextualised implementation science must be central to strengthening Africa’s clinical trial ecosystem.

## Bridging efficacy to effectiveness: The need for pragmatic and hybrid designs

Mash et al. appropriately differentiate between efficacy and effectiveness trials.^1^ We further suggest that hybrid effectiveness – implementation designs – which simultaneously assess clinical outcomes and implementation processes – are particularly suited to African settings, where understanding contextual barriers (e.g. health worker shortages, cultural acceptability, infrastructural deficits) is as important as evaluating clinical benefit. These hybrid models have shown value in primary care mental health interventions across sub-Saharan Africa^2^ and should now be mainstreamed in family medicine research.

## Expanding trial participation through decentralisation and digital innovation

The authors rightly highlight the limited participation of African primary care in global trials. One underexplored lever is decentralised clinical trials (DCTs) that use digital tools and community health infrastructure to enable recruitment, consent and data collection at the household or village level. Early pilots in West Africa during the coronavirus disease 2019 (COVID-19) pandemic demonstrated the feasibility of such models even in bandwidth-constrained environments.^3^ Investment in DCTs may also reduce urban-rural trial disparities, a long-standing equity concern.

## Community co-production and ethical governance

We support the call for robust ethical frameworks and emphasise that community co-production – not just engagement – must underpin trial design and execution. In contexts where historical research exploitation remains salient, ensuring transparent benefit-sharing, data sovereignty and community authorship rights is not only ethical but enhances trial relevance and sustainability.

## Conclusion

Mash et al.^1^ provide a vital foundation for early career researchers in family medicine. To advance their vision, we must integrate hybrid implementation methodologies, digital trial decentralisation and decolonised governance models. These steps will not only improve methodological rigour but also ensure that trials are grounded in the lived realities of African primary care systems.
